# Treatment of Cancer with Radio-Immunotherapy: What We Currently Know and What the Future May Hold

**DOI:** 10.3390/ijms22179573

**Published:** 2021-09-03

**Authors:** William Tyler Turchan, Sean P. Pitroda, Ralph R. Weichselbaum

**Affiliations:** Department of Radiation and Cellular Oncology and the Ludwig Center for Metastasis Research, University of Chicago, 5758 S Maryland Ave, Chicago, IL 60637, USA; wturchan@radonc.uchicago.edu (W.T.T.); Sean.Pitroda@bsd.uchicago.edu (S.P.P.)

**Keywords:** radiotherapy, immunotherapy, radio-immunotherapy, metastasis, oligometastasis

## Abstract

Radiotherapy and immunotherapy are most effective as cancer therapies in the setting of low-volume disease. Although initial studies of radio-immunotherapy in patients with metastatic cancer have not confirmed the efficacy of this approach, the role of radio-immunotherapy in patients with limited metastatic burden is unclear. We propose that further investigation of radio-immunotherapy in metastatic patients should focus upon patients with oligometastatic disease.

## 1. Introduction

Both radiotherapy and immunotherapy are effective as single therapies in select patients with cancer. While radiotherapy and immunotherapy differ in their indications, both treatments are most effective in patients with low-volume disease. Although adjuvant immunotherapy has been demonstrated to improve outcomes following radiotherapy in two disease sites, studies investigating the role of combined radiotherapy and immunotherapy in patients with metastatic disease have largely failed to demonstrate consistent and predictable efficacy. Patients with oligometastatic disease represent a subset of patients with metastatic cancer in whom disease burden is limited. These patients may be ideally situated to derive the greatest benefit from radio-immunotherapy. Thus, future studies utilizing radio-immunotherapy should focus upon patients with oligometastatic disease and should pursue novel means to widen the therapeutic ratio of radio-immunotherapy in this population.

## 2. Radiotherapy and Immunotherapy as Single Treatments: A Brief Overview

The utility of radiotherapy as a treatment for patients with cancer has been recognized for over a century [1]. Until recently the role of radiotherapy in the management of patients with metastatic disease has primarily been limited to the palliation of disease-related symptoms. However, recent data suggest that that radiotherapy may play a role in the definitive management of some patients with metastatic disease. Notably, the results of SABR-COMET, a phase II screening trial in which patients with a controlled primary tumor and ≤ 5 distant metastases (75% of patients had 1–2 metastases) were randomized to receive standard of care therapy with or without metastasis-directed ablative radiotherapy, showed that patients treated with radiotherapy had improved overall survival (OS) at five years compared to patients not receiving radiotherapy (42% vs. 18%, *p* = 0.006) [2]. Disease-site specific phase II randomized trials have similarly demonstrated improvements in outcomes with the addition metastasis-directed radiotherapy to standard management in patients with prostate cancer [3,4] and non-small cell lung cancer (NSCLC) [5,6]. Like SABR-COMET, these studies exclusively included patients with few distant metastases at the time of metastasis-directed radiotherapy. Although the results of ongoing confirmatory studies investigating the role of metastasis-directed radiotherapy in patients with few and several metastases separately (NCT03862911, NCT0372134, NCT02364557, NCT03137771) will provide further data, non-randomized series support the notion that low disease burden is an important predictor of long-term disease control in this population [7,8]. However, it is of note that, given some patients may require multiple interventions, OS may be a better measure of efficacy than progression-free survival (PFS) in this context.

Compared to radiotherapy, immunotherapy represents a relatively new strategy in the management of patients with cancer. Nonetheless the impact of immunotherapy has been dramatic among select populations of patients with metastatic disease. Notably, patients with metastatic melanoma treated with nivolumab and ipilimumab have recently been reported to have five-year OS of 52% [9], while the results of a recent investigation of pembrolizumab in patients with metastatic NSCLC with a programmed death-ligand 1 (PD-L1) tumor proportion score ≥ 50% demonstrate five-year OS of 30% and 25% in treatment-naive and previously treated patients, respectively [10]. These results are considerable improvements over historical results. Similar to radiotherapy, data support immunotherapy to be most efficacious in the setting of low-volume disease. Among patients treated for advanced melanoma with pembrolizumab on KEYNOTE-001, patients with less than the median baseline tumor size (summed diameter <10.2 cm) were noted to have improved overall objective response rate (ORR) (44% vs. 23%; *p* < 0.001) and OS (HR 0.38; *p* < 0.001) [11]. Thus, mounting data support the benefit of both radiotherapy and immunotherapy as monotherapies for patients with metastatic disease, especially in the setting of low disease burden.

## 3. What We Currently Know: Investigations of Combined Radio-Immunotherapy in Patients with Advanced Malignancies

### 3.1. Addition of Immunotherapy to Definitive Local Therapy

The results of a number of studies investigating various combinations of immunotherapy and radiotherapy in the definitive setting have recently been reported (Table 1). Among these studies, perhaps the best known is the PACIFIC trial, which randomized patients without progression of disease following definitive chemoradiotherapy for unresectable stage III NSCLC to adjuvant durvalumab or placebo beginning 1–42 days after the completion of chemoradiotherapy [12]. At four years, the addition of durvalumab significantly improved both PFS (35% [95% CI, 30–40%]) and OS (50% [95% CI, 45–54%] compared to placebo (20% [95% CI, 14–26%] and 36% [95% CI, 30–43%] for PFS and OS, respectively) [13]. Similarly, the recently reported CheckMate-577 trial, which randomized patients with Stage II-III esophageal and gastroesophageal junction cancer to receive adjuvant nivolumab or placebo 4–16 weeks following the completion of definitive therapy with neoadjuvant chemoradiotherapy followed by R0 resection, demonstrated improved median disease-free survival (DFS) among patients treated with nivolumab (22.4 months [95% CI, 16.6–34.0 months]), compared to those receiving placebo (11.0 months [95% CI, 9.3–14.3 months]) [14].

While the results of the PACIFIC and CheckMate-577 studies demonstrate the potential utility of immunotherapy to improve the outcomes of patients treated with radiotherapy for locally advanced malignancies, other attempts to combine radiotherapy and immunotherapy in the definitive setting have been less successful in demonstrating consistent benefits. The JAVELIN Head and Neck 100 and PembroRad trials both represent unsuccessful attempts to improve the outcomes of patients with locally advanced head and neck squamous cell carcinoma (HNSCC) with the addition of concurrent immune checkpoint blockade (ICB) agents to definitive radiotherapy [15,16]. Similarly, the results of CheckMate-498 and Checkmate-548, which investigated the addition of concurrent nivolumab to radiotherapy in patients with O6-methylguanine-DNA methyltransferase (MGMT)-unmethylated glioblastoma multiforme (GBM) and temozolomide and radiotherapy in patients with MGMT-methylated GBM, respectively, failed to demonstrate the efficacy of nivolumab in these settings [17,18,19,20,21]. The design and results of these studies are summarized in Table 1.

A number of potential explanations for the disparate results of studies combining radiotherapy and immunotherapy in the curative treatment of patients exist. Unlike unsuccessful trials in patients with HNSCC and GBM, the PACIFIC and CheckMate-577 trials utilized ICB in the adjuvant setting. Thus, it is possible that the inherent low volume of disease afforded by treatment in the adjuvant setting explains the difference in outcomes in the PACIFIC and CheckMate-577 trials compared to trials investigating radio-immunotherapy in patients with HNSCC and GBM. This hypothesis is bolstered by evidence that, as monotherapies, radiotherapy and immunotherapy have the greatest efficacy in patients with low-volume disease. The important benefits of adjuvant ICB in patients without evidence of residual disease following local therapy for high-risk melanoma [22,23] also support the theory that dissimilar outcomes of studies combining radiotherapy and immunotherapy in the definitive setting can be attributed to differences in disease burden. Moreover, it is of note that unlike tumors, which have been demonstrated to harbor radioresistant resident T-cells [24,25,26], T-cells in lymphoid organs, or circulating T cells, are known to be radiosensitive [25]. Draining lymph nodes have been demonstrated to facilitate anti-tumor immune response following radiotherapy by serving as sites of T-cell accumulation and priming [27]. Accordingly, studies using murine models treated with concurrent ICB and radiotherapy have demonstrated that the addition of the draining lymph nodes to the radiotherapy treatment volume results inferior OS [28]. Thus, it is conceivable that large elective nodal volumes contributed to the failure of studies investigating concurrent ICB and radiotherapy for locally advanced HNSCC [15,16].

### 3.2. Combined Radio-Immunotherapy in Patients with Metastatic Disease

Combined radio-immunotherapy has been investigated as a strategy for treating patients with metastatic disease. Unlike studies adding immunotherapy to radiotherapy as a means of increasing local response to radiotherapy and/or inducing anti-tumor immune effects against remaining subclinical disease, immunotherapy has primarily been investigated as a means of potentiating distant responses following radiotherapy in patients with metastatic disease. This paradigm stems from pre-clinical data demonstrating that abscopal effects at unirradiated tumor locations are mediated by T-cells [29] and that ICB prevents the development of distant metastases following radiotherapy [30]. Accordingly, a number of studies utilizing murine models have successfully induced abscopal responses following radiotherapy with the addition of ICB [31,32,33].

In contrast to the pre-clinical setting, meaningful abscopal responses are rather uncommon clinically [34]. A notable exception comes from a study reported by Golden et al. in which patients with ≥3 metastases were treated with radiotherapy to one metastasis (35 Gy in 10 fractions) in combination with granulocyte-macrophage colony-stimulating factor (GM-CSF) [35]. Abscopal responses were observed in 11 of 41 enrolled patients. However, the majority of abscopal responses (defined as a decrease in the longest diameter of any measurable non-irradiated lesion by ≥30%) were rather modest [35]. It is also of note that abscopal responses primarily occurred in patients with relatively low pre-treatment disease burden in this study. Of the 11 patients in whom abscopal responses were observed, eight patients had three metastases at study enrollment (the minimum allowed) and three patients had 4–6 metastases. Further, no patients with >6 metastases experienced abscopal responses [35]. The benefit of adding metastasis-directed radiotherapy to immunotherapy has been the subject of a number of comparative phase I/II trials, the designs and results of which are summarized in Table 2. These studies represent a variety of histologies, including NSCLC [36,37,38,39], melanoma [38], HNSCC [40], and adenoid cystic carcinoma (ACC) [41]. With the exception of the report by Curti et al. in which patients received interleukin-2 (IL-2) three days after treatment with either 20 Gy or 40 Gy given in 20 Gy fractions [38], patients in these studies were treated with an anti-PD-1/PD-L1 agent [36,37,38,39,40,41]. Patients treated with radiotherapy in these studies were largely treated with radiotherapy doses of 24–30 Gy given in 3–5 fractions [36,37,40,41], with exceptions in the study reported by Welsh et al., in which patients were treated with 48 Gy in four fractions if deemed clinically feasible (*n* = 19) and otherwise received 45 Gy in 15 fractions (*n* = 21) [39], and the previously described study reported by Curti et al. Among patients treated with radiotherapy in these studies, radiotherapy was delivered before [38], following [36,37,41], between cycles [40], and concurrently [39], relative to ICB. Despite the differences among these studies, a similarity exists in that all failed to demonstrate significant improvements in ORR, PFS, and OS with the addition of radiotherapy to immunotherapy [36,37,38,39,40,41]. It is of note that, in an unplanned, pooled analysis of the studies by Theelen et al. and Welsh et al., patients treated with radiotherapy had improved PFS (4.4 vs. 9.0 months, *p* = 0.045) and OS (8.7 vs. 19.2 months, *p* < 0.001) [42]. On the whole, the results of comparative studies of radio-immunotherapy suggest that previously tested strategies for combining radiotherapy and immunotherapy rarely induce clinically meaningful abscopal responses in patients with metastatic disease.

While clinical studies investigating radio-immunotherapy in patients with metastatic disease have largely failed to demonstrate improved outcomes compared to treatment with immunotherapy alone, a number of factors may explain these results. Although comparative studies investigating the addition of radiotherapy to immunotherapy have been negative regardless of whether a single [36,37,40] or multiple metastases [38,39,41] were targeted with radiotherapy, it is of note that all of these studies have required at least one metastasis to be remain unirradiated. In contrast to these studies, in a non-randomized prospective study of NSCLC patients with ≤ 4 metastases (93% had ≤ 2 metastases and 61% had one metastasis) reported by Bauml et al., locally ablative therapy to all sites of distant metastasis in combination with pembrolizumab yielded impressive gains over historical outcomes, including a two-year PFS of 56% among patients metachronous disease [43]. Pre-clinical data also support the idea that radio-immunotherapy may be most effective when all sites of distant metastasis are targeted with radiotherapy [44]. Radiotherapy is capable of increasing tumor T-cell infiltration and killing resistant tumor clones, while sparing radioresistant resident T-cells [24,25,26]. Moreover, radiotherapy enhances major histocompatibility complex class I (MHC-I) expression [45], down regulation of which is a known mechanism of tumor immune evasion [46], and induces/up-regulates immunogenic genes [47] that may to drive response to ICB [48]. Additionally, it is possible that, similar to the adjuvant setting, radio-immunotherapy is most effective in the setting of low-volume disease. Unlike comparative studies of immunotherapy with and without radiotherapy, in which the majority of patients had relatively high-burden metastatic disease, the studies reported by Golden et al. [35] and Bauml et al. [43], respectively, primarily enrolled patients with low-volume disease. Thus, it is plausible that the failure of comparative studies to demonstrate the efficacy of radio-immunotherapy in metastatic patients can, in part, be attributed to the relatively high average disease burden of patients in these studies.

## 4. What the Future May Hold: Strategies to Maximize the Therapeutic Ratio of Radio-Immunotherapy in Cancer Patients Going Forward

Overall, studies investigating the utility of radiotherapy and immunotherapy in cancer patients suggest that these therapies are most efficacious, both individually and in combination, in the setting of low-volume disease. Moreover, data support the hypothesis that radio-immunotherapy is most effective when all sites of macroscopic disease are treated with radiotherapy and potentially immunosuppressive nodal irradiation is avoided. Given these criteria, patients with oligometastatic disease may be expected to derive the greatest potential benefit from radio-immunotherapy. Thus, future studies of radio-immunotherapy should focus upon this population.

The oligometastatic hypothesis, which was initially proposed in 1995 [49], predicts the existence of a state in which patients have a low volume of metastatic disease that is unlikely to progress rapidly [49]. Given that patients with oligometastatic disease inherently have a low disease burden, these individuals may be predilected to derive the greatest benefit from radio-immunotherapy. Moreover, because patients with oligometastatic cancer are more likely to have disease that can feasibly be comprehensively covered in radiotherapy treatment volumes without the need for potentially immunosuppressive large-volume nodal irradiation, these patients may be especially likely to benefit from radio-immunotherapy. As a result, future studies should be conducted to determine the role of radio-immunotherapy in the management of patients with oligometastatic cancer.

Since it was first described, the oligometastatic hypothesis has been refined in a number of ways. In addition to the volume and rate of progression, which define the oligometastatic state, a number of other features, including disease histology, lymph node status, and timing of metastasis, have been demonstrated to be prognostic in this population [50,51,52]. Thus, these factors are likely to also be important considerations in future studies of radio-immunotherapy in patients with metastatic disease. Moreover, molecular features predictive of oligo- versus poly-metastatic progression have been identified [52] and can be used to stratify patients by risk of failure following metastasis-directed local therapy [53]. Thus, future studies should utilize molecular information to identify patients most likely to benefit from radio-immunotherapy. In addition to focusing on patient populations most likely to benefit from radio-immunotherapy, future studies should also investigate novel means of widening the therapeutic ratio of radio-immunotherapy. A number of ongoing studies aim to characterize combinations of immunomodulatory agents that may be able to enhance and/or improve upon the efficacy of radio-immunotherapy regimens that utilize ICB [54]. Recent evidence also suggests that the microbiome affects anti-tumor immune responses induced by both ICB [55] and radiotherapy [56,57], and therefore this could be a point of further investigation going forward.

In conclusion, radiotherapy and immunotherapy have been shown to improve outcomes in distinct patient populations, though both therapies are seemingly most effective in patients with low-volume disease. Unlike the addition of adjuvant immunotherapy following radiotherapy, which has improved outcomes in multiple disease sites, initial studies of radio-immunotherapy in metastatic patients have not confirmed the efficacy of this approach. However, additional studies are needed to determine the role of radio-immunotherapy in patients with oligometastatic disease, given that these individuals inherently have a limited metastatic burden and may be most likely to reap the benefits of combined radiotherapy and immunotherapy.

## Figures and Tables

**Table 1:** Studies of radiotherapy with and without immunotherapy in patients with locoregionally advanced cancer.

Study	Population	Design	Results
PACIFIC [13]	*n* = 713, Unresectable Stage III NSCLC ^a^	Definitive chemo-RT ± adjuvant durvalumab starting 1–42 days later	Median follow-up: 34.2 mo PFS: HR 0.55 [95% CI 0.44–0.67] OS: HR 0.71 [95% CI 0.57–0.88]
CheckMate-577 [14]	*n* = 794, Stage II-III Esophageal/GEJ	Neoadjuvant chemo-RT and R0 resection ± adjuvant nivolumab starting 4–16 weeks later ^b^	Median follow-up: 24.4 mo DFS: HR 0.69 [95% CI 0.56–0.86]
JAVELIN H&N 100 [15]	*n* = 697, LA HNSCC	Definitive chemo-RT ± concurrent and adjuvant avelumab	Median follow-up: 14.6 mo PFS: HR 1.21 [95% CI 0.93–1.57] OS: HR 1.31 [95% CI 0.93–1.85]
PembroRad [16]	*n* = 131, LA HNSCC	Definitive concurrent RT + cetuximab vs. RT + pembrolizumab	Median follow-up: 25 mo PFS: HR 0.83 [95% CI 0.53–1.29] OS: HR 0.83 [95% CI 0.49–1.40]
CheckMate-498 [17]	*n* = 560 MGMT-um GBM	Definitive concurrent RT + TMZ vs. RT + nivolumab	PFS: HR 1.38 [95% CI 1.15–1.65] ^c^ OS: HR 1.31 [95% CI 1.09–1.58] ^c^
CheckMate-548 [18]	*n* = 693, MGMT-m GBM	Definitive concurrent RT + TMZ ± concurrent nivolumab	PFS: NS [19] OS: NS [20]

NSCLC = non-small-cell lung cancer, chemo-RT = chemoradiotherapy, mo = months, PFS = progression-free survival, HR = hazard ratio, 95% CI = 95% confidence interval, OS = overall survival, GEJ = gastroesophageal junction, R0 resection = microscopically margin-negative, DFS = disease-free survival, H&N = head and neck, LA HNSCC = locally advanced head and neck squamous cell carcinoma, RT = radiotherapy. MGMT-um = O6-methylguanine-DNA methyltransferase-unmethylated, GBM = glioblastoma multiforme, TMZ = temozolomide, MGMT-m = O6-methylguanine-DNA methyltransferase-methylated, NS = non-significant. ^a^ In order to be eligible for the PACIFIC trial, patients had to have no evidence of disease progression following definitive chemoradiotherapy. ^b^ In order to be eligible for CheckMate-577 patients had to have residual pathologic disease at the time of resection. ^c^ Preliminary results of CheckMate-498 are available on ClinicalTrials.gov; Identifier: NCT02617589 [21].

**Table 2:** Studies of immunotherapy with or without radiotherapy in patients with metastatic cancer.

Author, Year	Population	Design	A(RT + ICB VS. ICB) a
Moreno et al., 2018 [36]	*n* = 20/33, ^b^ Met NSCLC	Cemiplimab ± 9 Gy × 3 to 1 lesion; RT given within 1 week of ICB	18% vs. 40% (NS)
Theelen et al., 2019 c [37]	*n* = 76, Met NSCLC	Pembrolizumab ± 8 Gy × 3 to 1 lesion; RT given within 1 week of ICB	36% vs. 18% (*p* = 0.07)
Curti et al., 2020 [38]	*n* = 44, Met Melanoma	IL-2 ± 20 Gy × 1–2 to 1–3 lesions; RT given 3 days prior to IL-2	54% vs. 35% (NS)
Mcbride et al., 2020 [40]	*n* = 62, Met HNSCC	Nivolumab ± 9 Gy × 3 to 1 lesion; RT given between cycles 1 and 2 of ICB	29% vs. 35% (*p* = 0.86)
Welsh et al., 2020 c [39]	*n* = 20/80, ^d^ Met NSCLC	Pembrolizumab ± RT ^e^ to 1–4 lesions; RT given concurrent with cycle 1 of ICB	22% vs. 25% (*p* = 0.99)
Mahmood et al., 2021 [41]	*n* = 20, Met ACC	Pembrolizumab ± 6 Gy × 5 to 1–5 lesions; RT given within 1 week of ICB	50% vs. 70% (*p* = 0.65)

RT = radiotherapy, ICB = immune checkpoint blockade, Met = metastatic, NSCLC = non-small-cell lung cancer, Gy = Gray, NS = non-significant, IL-2 = interleukin-2, HNSCC = head and neck squamous cell carcinoma, ACC = adenoid cystic carcinoma, ^a^ Out-of-field objective response rates for patients treated with radiotherapy and immunotherapy and immunotherapy alone, respectively. ^b^ The report by Moreno et al. includes the results of two separate non-randomized phase I expansion cohorts in which patients were treated with radiotherapy and pembrolizumab and pembrolizumab alone, respectively. ^c^ An unplanned pooled analysis of the studies by Theelen et al. and Welsh et al. was performed and demonstrated improved progression-free survival (4.4 vs. 9.0 months, *p* = 0.045) and overall survival (8.7 vs. 19.2 months, *p* < 0.001) with the addition of radiotherapy to pembrolizumab compared to pembrolizumab alone [42]. ^d^ The study reported by Welsh et al. included 20 patients in the initial phase 1 component. 80 patients randomized in the subsequent phase II portion. ^e^ Patients randomized to randomized to pembrolizumab and radiotherapy in the study reported by Welsh et al. were treated with a radiotherapy dose of 12.5 Gy × 4 if deemed clinically feasible (*n* = 19) and otherwise were treated with 3 Gy × 15 (*n* = 21).
